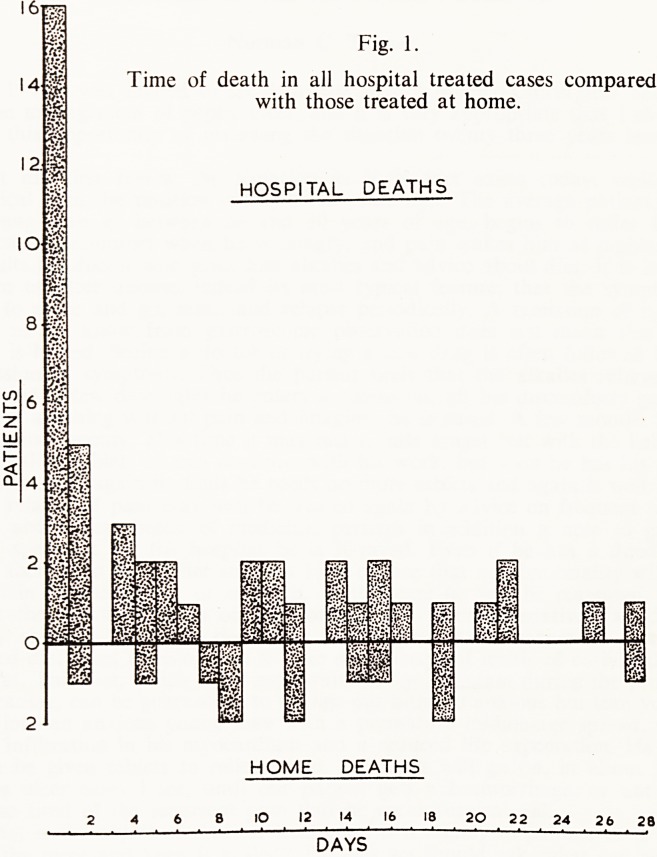# Home Care of Myocardial Infarction

**Published:** 1969-10

**Authors:** H. G. Mather


### HOME CARE OF MYOCARDIAL INFARCTION

H. G. Mather

Since I am not a graduate of Bristol University, I had no first-hand opportunity of appreciating the worth of Professor Bruce Perry as a teacher and diagnostician. Very soon after my appointment here 13 years ago, I realized these abilities and also his critical and self-critical approach to the so called advances in medicine, a good example being anti-coagulant treatment. The study which I am about to report is a comparison over two years of hospital and home treatment of myocardial infarction, and I here wish to acknowledge the co-operation of colleagues in general and hospital practice, many of them trained by Bruce Perry.

Many patients and indeed doctors express a wish to be treated at home when afflicted by this disease, yet there are as yet no published figures on home care. Four years ago my general practitioner colleague John Wright conducted a retrospective survey of infarcts in practices in the South West and found that approximately half were treated at home; the mortality rate was similar to those admitted to hospital. By then the Intensive Care Unit at Southmead Hospital had been established, and we felt it desirable to conduct a prospective study comparing the fate of the patients treated at home with those cared for in an Intensive Care Unit and general wards of a district general hospital (Southmead). About half the practitioners in the area agreed to co-operate and the expenses of the research were covered by the Ministry of Health. The plan of the trial was agreed by a central committee chaired by Sir Robert Platt, with statistical help from Professor Archibald Cochrane, and Miss E. H. L. Duncan from the Department of Public Health.

The possible difficulties of home nursing of women made us confine our study to males under 70 years of age. Ethical considerations made it essential for the general practitioner to be able to decide whether an individual should be admitted or kept at home. Thus if he saw a patient suspected of having an infarct, he had three choices:—

1) he could send the patient forthwith to the hospital of his choice for treatment by the appropriate clinical team. Inevitably there was some variation in medical care of these patients—*The Elective Hospital Group.*
2) he could for medical or social reasons decide to treat the patient at home, with help in diagnosis from the research registrar who took E.C.G.'s and blood tests. If any change in the clinical condition occurred which made him feel the patient should be admitted, this was always possible, but the patient remained in his orginal *Elective Home Group.*
3) where in his opinion the patient could equally well be treated at home or in hosiptal, he opened a sealed envelope containing instructions which had previously been allocated on a random basis. When these indicated hospital, the patient was admitted to the Intensive Care Unit at Southmead Hospital, under my care, and after 48 hours he was, if fit, transferred to the adjacent medical ward. This was the *Random Hospital Group.* When the slip indicated home care, the patient was cared for by his doctor with the research registrar again helping in the diagnosis. This was the *Random Home Group.*

The general practitioner also notified us of any sudden deaths in his practice due to myocardial infarction so that we could gauge the proportion of treatable patients.

### CRITERIA FOR ACCEPTANCE

Diagnositic criteria for myocardial infarction vary throughout the world, thus making comparison of statistics difficult. For this study, we had rigid standards which inevitably meant rejection of some patients after allocation to the different groups. We here discuss only those who had in the previous forty-eight hours a history of cardiac pain together with

1) evolution of the classical injury current on E.C.G. (W.H.O. criteria 1 Ae — World Health Organisation Technical report series No. 168, Geneva, 1959)
2) typical Q and T wave changes in association with a significant rise in the appropriate serum enzyme test (W.H.O. criteria 1 Aa to 1 Bo)
3) subsequent post mortem evidence of infarction.

### TREATMENT

The elective hospital group was treated according to the decisions of the staff in the hospital, but about nine tenths were in Southmead and received treatment from my colleagues or myself similar to those in the random group. All these were kept in the intensive care unit for a minimum of 48 hours during which their E.C.G. was monitored. An intravenous glucose drip was maintained so that drugs could be given rapidly. Lignocaine was given for multiple or multifocal extra systoles, heroin or morphia in small but adequate doses for pain. Digoxin was used for supraventricular arrythmias, and for the early signs of heart failure together with frusemide. Atropine was exhibited for bradycardia, steroids and isoprenaline for heart block with occasional transvenous pacing. Ventricular fibrillation was treated by immediate DC countershock. No patient received anticoagulants as a routine.

The usual period of bed rest was three weeks, with a fourth week of graded activity in the ward. At all times bedside commodes were used, the patient washed and fed himself, and performed leg, arm, and breathing exercises. Smoking was not allowed.

### NUMBERS

In the first two years of our study, 170 general practitioners notified us of 496 patients suspected of having had myocardial infarctions; of these only 30 proved to be false diagnoses—a tribute surely to their acumen. Seventy nine had to be rejected as they failed to fufil our diagnositic criteria, and are classed here as coronary insufficiences. This left 387 for study and of these 34 were sudden deaths in patients not seen by a doctor; all were confirmed by necropsy. Two patients sent in to hospital for other reasons were found to have infarcts, and one died shortly after the doctor's arrival, thus leaving 350 who were allocated to the different groups. The largest group was of course "elective hospital" (191) comprising patients whose home arrangements (96) made admission desirable, or where the doctor judged that for medical reasons he should be in hospital. A few in this group were admitted direct from their place of work, or by locums. Sixty patients were in the "elective home" group, 50 in the "random hospital", and 49 in the "random home" groups.

| AGE OF PATIENTS | <50 | 50–59 | 60–69 |
|---|---|---|---|
| RANDOM HOME | 10 % | 50 % | 40 % |
| RANDOM HOSPITAL | 24 % | 48 % | 28 % |
| ELECTIVE HOME | 18 1/3 % | 43 1/3 % | 38 1/3 % |
| ELECTIVE HOSPITAL | 20 % | 35 % | 45 % |

TABLE I
Age distribution of patients in the four treatment groups.

| SOCIAL CLASS | I & II | III | IV & V | N. S. |
|---|---|---|---|---|
| RANDOM HOME | 20½ % | 61 % | 18 % | |
| RANDOM HOSPITAL | 18 % | 62 % | 20 % | |
| ELECTIVE HOME | 20 % | 41 2/3 % | 36 2/3 % | 1 2/3 % |
| ELECTIVE HOSPITAL | 25 % | 46 % | 27 % | 2 % |

TABLE II
Analysis of social class according to the Registrar General's classification. N.S.: not specified, i.e. inadequate data available.

The age distribution of the patients is shown in Table I where it will be seen that no gross variations in the groups occurred, though it happened with these relatively small numbers that rather more elderly patients appeared in the random home group than the random hospital. Analysis by social class according to the Registrar General's classification is shown in Table II from which it will be seen that there are no gross differences. In particular there was no tendency for the more intelligent and wealthy to be kept at home by choice.

### GRADING OF SEVERITY

An attempt was made to grade severity both at first contact with a doctor and subsequently. This is fraught with difficulties because of varying standards of observation, and inevitably the hospital groups have a worse grade because of their more frequent examination. The grades were:—

1) no sign of heart failure or hypotension (defined as systolic blood pressure below 100 mm.Hg).
2) normotensive with signs of heart failure: persistent basal crepitations in the absence of respiratory disease or any two or (a) dyspnoea, (b) tachycardia greater than 100/min, (c) raised J.V.P. (d) oedema, (e) gallop rhythm.
3) hypotension with or without confusion, restlessness, pallor, cyanosis, sweating, cold skin.
4) hypotension with signs of heart failure.

SEVERITY AT FIRST CONTACT

| | 1 | 2 | 3 | 4 | N.S. |
|---|---|---|---|---|---|
| RANDOM HOME | 78 | 10½ | 5 | 4 | 2½ % |
| RANDOM HOSPITAL | 68 | 16 | 14 | | 2 % |
| ELECTIVE HOME | 66⅔ | 13⅓ | 10 | | 6 % |
| ELECTIVE HOSPITAL | 52 | 24 | 9 | 3 | 12 % |

TABLE III

Grading of severity in percentages when first seen by a doctor.
N.S.: not specified.

| FINAL SEVERITY | 1. | 2. | 3. | 4. | N.S. |
|---|---|---|---|---|---|
| RANDOM HOME | 32 | 24 | 14 | 28 | 2 % |
| RANDOM HOSPITAL | 22 | 22 | 10 | 46 | |
| ELECTIVE HOME | 38 ⅓ | 25 | 11 ⅔ | 16 ⅔ | 8⅓ % |
| ELECTIVE HOSPITAL | 20 | 28 | 16 | 36 | |

TABLE IV

Severity grading according to the worst stage reached during treatment.

The grading at first contact with a doctor is shown in Table III where it is seen that 12% of the elective hospital group were "not specified" that is inadequate clinical data were available. The worst grade a patient ever reached, or final grading, is shown in Table IV; a higher proportion of hospital patients have been classified in groups 3 and 4 partly because of more frequent observations and possibly because of deterioration caused by being admitted.

### MORTALITY

The only important and objective assessment of treatment in this disease comes from comparing mortality rates. Table V shows that the overall death rate in the first 28 days for those who survived long enough to see a doctor is 16.5%. The really important observation is that the mortality in the random home group (9%) was less than the random hospital (16%). Although these numbers are as yet not statistically significant, they do indicate that home treatment is not only ethically justifiable, but may even be preferable. Careful analysis of the complications such as heart block and ventricular dysrhythmias is being undertaken but time does not allow for their description.

When the time of death of the combined hospital groups is recorded it will be seen (Table VI) that there is the same high mortality in the first 48 hours as reported by most authors. In the home treated cases however, the deaths occurred in a scattered fashion during the first month, with no early peak. This is shown graphically in Fig. 1 where the total hospital and home groups are compared.

### MORTALITY RATES

| | CASES | DEATHS | % | SECONDARY HOSPITAL | DEATHS |
|---|---|---|---|---|---|
| OVERALL | 387 | 95 | 25 | | |
| TREATED CASES | 350 | 58 | 16·5 | | |
| RANDOM HOME | 49 | 4 | 9 | 7 | 1 |
| RANDOM HOSPITAL | 50 | 8 | 16 | | |
| ELECTIVE HOME | 60 | 8 | 13·3 | 15 | 4 |
| ELECTIVE HOSPITAL | 191 | 38 | 20 | | |

TABLE V

Mortality rates in the treatment groups. The overall mortality includes the sudden deaths before being seen by a doctor which occurred during the two years' study. The "secondary hospital" patients were those who were subsequently admitted to hospital after original treatment at home.

| TIME OF DEATH | TOTAL | HOURS 0–24 | HOURS 24–48 | DAYS 2–7 | DAYS 8–28 |
|---|---|---|---|---|---|
| RANDOM HOME | 4 | | | | 4 |
| RANDOM HOSPITAL | 8 | 2 | 2 | 1 | 3 |
| ELECTIVE HOME | 8 | | 1 | 1 | 6 |
| ELECTIVE HOSPITAL | 38 | 14 | 3 | 7 | 14 |

TABLE VI

Time o[illegible] death from the estimated onset of the myocardial infarct.

Fig. 1.

Time of death in all hospital treated cases compared with those treated at home.

### CONCLUSIONS

This study, which is still proceeding, shows that home treatment still plays a part in the management of myocardial infarction, in males under 70, when domestic arrangements are saisfactory. The mortality in randomly selected groups shows a lower rate for home compared with hospital, though the difference is not as yet statistically significant.

*Acknowledgement*

I wish to thank all my colleagues who have helped in this study, especially Dr. Conrad Guerrier who as research registrar was responsible for the collection and analysis of most of the data.

## Figures and Tables

**Fig. 1. f1:**